# Restricting E‐Cigarette Flavour Names to Reduce Youth Appeal: An Analysis of New Zealand's 2024 Regulations

**DOI:** 10.1002/hpja.70171

**Published:** 2026-03-16

**Authors:** Lucy Hardie, Cohen Radich, Meghana Bandlamudi, Judith McCool

**Affiliations:** ^1^ School of Population Health The University of Auckland Auckland New Zealand; ^2^ Faculty of Engineering and Design The University of Auckland Auckland New Zealand

**Keywords:** adolescent, electronic nicotine delivery systems, health policy, marketing, vaping

## Abstract

**Issue Addressed:**

Appealing e‐cigarette flavours have been an important factor in experimentation and uptake among young people. In an effort to curb high rates of youth vaping, in 2024, the New Zealand Government introduced regulations to restrict e‐cigarette flavours that may appeal to young people. The regulations require that e‐cigarette products are described using up to two flavour names from a specified list. This research analyses whether online retailers comply with the regulations and provides insights into popular flavours.

**Methods:**

Using content analysis methods, we identified popular online retailers in New Zealand. We extracted flavour names from e‐cigarette product listings on retailer websites to analyse compliance and categorised products to explore popular flavour profiles.

**Results:**

Of the 22 retailers included in the sample, only 4 (18.0%) fully complied with the restrictions. Of the 6939 total e‐cigarette products (e‐liquids, pods and disposables), 25.9% (*n* = 1794) did not comply with the regulations. Most flavours in the sample were fruity or sweet (78.9%).

**Conclusion:**

New Zealand retailers frequently breach the flavour regulations. Despite many permitted options, retailers often attempt workarounds to bypass regulations, including listing products under previous names or descriptors that are not permitted, such as ‘ice’ or ‘cola’. Sweet and fruit flavours dominate the online market.

**So What?:**

E‐cigarette flavour restrictions should include sweet and fruit flavours to reduce appeal to youth and be supported by rigorous enforcement to prevent common retail workarounds.

## 1 Background

Youth vaping has emerged as a significant public health concern globally. Multiple factors have driven uptake, including wide availability, aggressive youth‐related marketing and design developments. Flavours are also an important factor in the appeal of e‐cigarettes among young people [1, 2], particularly sweet or fruity flavours [3]. Sweet flavours may also reduce perceptions of harm among teenagers [4] and minimise bitterness, increasing e‐cigarette palatability [5].

Several jurisdictions have introduced restrictions on flavours to reduce or prevent youth uptake, and these have been implemented in various ways. For example, in the United States, California and Ohio banned all flavoured e‐cigarette products in 2022 [6, 7]. Australia banned all flavours except for tobacco, mint, and menthol in 2024 [8], similar to other countries, including Estonia and Denmark [9, 10]. In 2024, the Netherlands restricted e‐cigarette flavours to tobacco and unflavoured, introducing a limited list of permitted flavour additives to ensure the industry cannot circumvent the restrictions by labelling products as tobacco whilst incorporating palatable additives to enhance taste [11].

Since 2020, New Zealand has had certain restrictions on flavours, including a rule whereby products sold by general retailers, such as convenience stores and gas stations, may only sell mint, menthol, and tobacco flavours [12]. However, Specialist Vape Retailers (SVRs), which are registered, dedicated e‐cigarette stores, were, until recently, allowed to stock a full range of flavours. Despite these and other restrictions, such as advertising bans and minimum purchase age introduced in 2020, rates of use continued to increase rapidly, with daily use among 15–24 year olds increasing from 4.3% to 22.1% between 2019 and 2023 [13].

In response to the rising rates of vaping among young people, the New Zealand Government introduced regulations in 2023 to restrict permitted flavour names, in an effort to reduce the appeal of these products to young people [14]. The new regulations stipulate that retailers must describe the ‘actual flavour of the vaping product using only one or two flavour names from Schedule 4A’ See Table 1. The regulations sought to reduce flavour descriptors with youth appeal, such as gummy‐bear, candy, and soda flavours, including cola and lemonade [16].

**TABLE 1:** Permitted e‐cigarette flavour names in New Zealand (retailers can select up to two) [15].

Category	Flavour name (retailers may select up to two words)
Tobacco	Tobacco
Mint	Menthol, Mint, Peppermint, Spearmint
Nuts and grains	Almond, Hazelnut, Nut, Oat, Peanut, Pecan
Spice	Cinnamon, Clove, Licorice, Nutmeg, Pepper, Spice
Coffee/tea	Cappuccino, Coffee, Espresso, Latte, Tea
Fruit	Apple, Banana, Berry, Blackberry, Blueberry, Cherry, Citrus, Coconut, Grape, Guava, Kiwifruit, Lemon, Lime, Lychee, Mango, Orange, Passionfruit, Peach, Pear, Pineapple, Plum, Pomegranate, Raspberry, Strawberry, Tropical, Watermelon
Sweet and sour	Caramel, Chocolate, Cream, Custard, Honey, Sour, Sweet, Vanilla
None	Unflavoured

Flavours are a key component of the appeal of e‐cigarette products and, therefore, profits. As such, the industry has actively opposed proposed flavour restrictions [7], including in New Zealand. For example, British American Tobacco in its 2020 submission to the New Zealand Parliament stated that e‐cigarette flavours are important to support smokers to transition away from smoking and that ‘Barriers to this access for adults will have unintended consequences of continued or greater smoking incidence’ [17]. Given the industry's opposition to flavour restrictions, understanding how retailers respond to such regulations is central for evaluating policy impact and guiding future interventions.

This study aimed to evaluate the e‐cigarette flavour‐naming regulations, which specify permitted words for describing each product's flavour. Our first objective was to determine whether online retailers complied with the restrictions 9 months after the regulations were enacted. The next objective was to describe the proportion of total e‐cigarette products that adhered to or violated the prescribed flavour naming restrictions. A further objective was to identify the most prominently featured or marketed e‐cigarette flavours to understand consumer preferences. This research contributes to the evidence base informing regulatory approaches to protect youth from vaping by examining compliance and flavour trends.

## 2 Methods

### 2.1 Retail Sample

Between October and December 2024, we conducted a structured keyword search to identify online retailers using methods identified in previous research [16]. We ran the series of keyword searches in a Google Chrome incognito window and included retailers listed on the first two pages of Google results. Retailers were included if they were New Zealand‐based, as defined by a New Zealand domain name, and identified as dedicated e‐cigarette retailers.

### 2.2 Data Collection

E‐cigarette products generally fell into four categories: prefilled products, including disposables and pods, and e‐liquid vials, typically available as nicotine salts or freebase nicotine. For each category, we first sorted the items by popularity using the website sort tool, for example, ‘best‐selling’ or ‘popular’. Flavour names were then manually extracted into Microsoft Excel v16.99 for analysis. In our study, we included the text describing flavours for each item, as presented for sale on the retailer websites.

### 2.3 Analysis

#### 2.3.1 Retailer Compliance and Age Verification

We first assessed each retailer individually, recording if they stocked non‐compliant products. If they only stocked permitted products, they were recorded as fully compliant.

#### 2.3.2 Flavour Analysis—Non‐Compliant Items

The New Zealand regulations of 2024 stipulate that retailers can use two words from an approved list to describe e‐cigarette product flavours (Table 1). We assessed the compliance of each item by assessing whether (a) more than two words were used to describe the flavour (yes/no), and (b) if the descriptor text was approved under the regulations (yes/no). If the item met either of these criteria, it was marked as non‐compliant. Non‐compliant items were then extracted for further analysis.

We categorised the non‐compliant items using an iterative process to assess the main ways retailers were breaching the regulations, identifying common strategies. We organised breaches into categories using an iterative process to identify similarities.

#### 2.3.3 Flavour Analysis—Compliant Products

After removing non‐compliant flavours, we assessed the most frequently listed flavours on the New Zealand market. This was done by counting each instance of an approved flavour descriptor in the sample of compliant flavours. Flavours were categorised into groups aligned with the regulations. For example, flavour descriptors such as apple and strawberry fall under the fruit category.

## 3 Results

### 3.1 Sample of Retailers and Products

We identified 22 popular e‐cigarette retailers for inclusion (Table S1). We extracted flavour names from 6939 e‐cigarette products (e‐liquids, pods and disposables). Retailers stocked between 6 and 1683 flavoured products (median = 160).

### 3.2 Total Product Compliance

We found that, 9 months after enactment, 25.8% (*n =* 1794) of the 6939 flavoured e‐cigarette products and liquids were not compliant with New Zealand flavour‐naming regulations.

### 3.3 Retailer Compliance

Four of the 22 retailers were fully compliant with the flavour name regulations. The remaining 18 (81.8%) displayed between 1 and 496 non‐compliant products (median = 18) on their websites.

### 3.4 Common Compliance Breaches and Attempts to Bypass Flavour Restrictions

Of the 1794 products which breached regulations, the word ‘ice’, which is generally used to describe the cooling sensation similar to menthol, was used in 455 products (25.4% of the breaches). Banned soda flavours, such as ‘Cola’ and ‘Lemonade’ and candy flavours, such as ‘Cotton Candy’ and ‘Gummy Bear’ (*n* = 206, 11.4%) were also identified. In other examples, retailers used colours or other descriptors in parentheses to differentiate between products, for example, ‘Tobacco (Gold)’. Other retailers used fabricated flavour descriptions such as ‘Iced Rain Bops’. In 162 instances (9.0% of the breaches), more than two flavours were utilised to describe products, for example, Strawberry Melon Apple, where only two flavour descriptors are permitted.

The majority of breaches (*n* = 1346, 75.0%) resulted from retailers' use of prohibited flavour names previously used to describe products. They did this using a combination of methods. For example, by presenting the permitted name followed by text that stated what the product was previously called, for example, ‘Blueberry Raspberry renamed from Blue Razz Lemonade’ [18] or by using the abbreviation ‘AKA’ to indicate ‘also known as’, for example, ‘Sweet Blueberry AKA Blue Cotton Candy’ [19]. Other retailers used brackets, for example, ‘Sweet Vanilla (ex cola)’ [20]. Other retailers included a flavour as part of the brand name, for example, ‘Vapetasia Lemonade Salts’ followed by the compliant flavour name ‘Raspberry Lemon’ [21] or ‘Mr Wicky Sherbert – Berry Grape’ [22]. These strategies attempt to bypass the regulations by utilising flavours with youth appeal, such as soda and candy, which are not permitted. In other cases, the website text used a compliant name, but the product image displayed the original (prohibited) name. E‐cigarette product images were prohibited in June 2025 [23].

### 3.5 Most Popular Flavours

To identify the most popular flavours on each website, we used the ‘sort’ function to list products as ‘best‐selling’ or ‘most popular’ across the categories on each website. We included the 10 most popular flavours for each category (disposables, freebase, nicotine salt, etc.), resulting in a subset of 441 products. 46.5% of products in the subset (*n* = 205) did not comply with the regulations. Because non‐compliant descriptors often contain non‐standard words to describe flavours, we categorised them broadly, as sweet or fruit flavoured, 58.0% (*n* = 119), candy, soda or dessert, 17.6% (*n* = 36), tobacco, 12.2% (*n* = 25), mint, 8.3% (*n* = 17) and other, 4.8% (*n* = 10).

**FIGURE 1:**
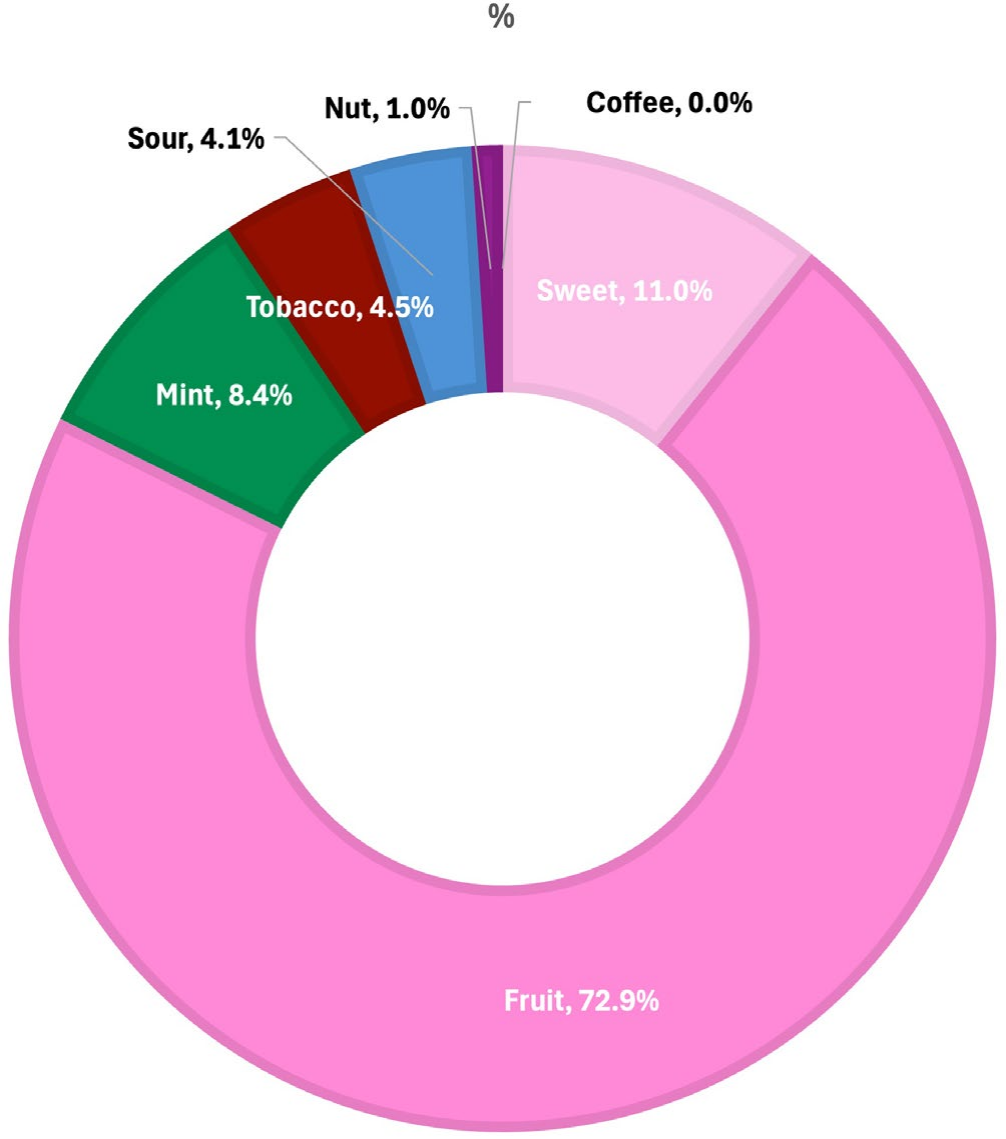
Most popular flavours (as reported by retailers) *n* = 441.

The compliant descriptor words included in this subset of the most popular flavours totalled 785 (as more than one flavour descriptor is often used). The most popular flavour descriptors were Berry (*n* = 98), Grape (*n* = 58), Apple (*n* = 56), Mango (*n* = 42) and Mint (*n* = 41). When organised into regulatory categories, the most popular flavour category was Fruit (72.9%; *n* = 572), of which more than half (56.2%) were berry flavours. The next most popular category was Sweet (Caramel, Chocolate, Cream, Custard, Honey, Sweet or Vanilla) at 11.0% (*n* = 86), followed by Mint at 8.4% (*n* = 66) and Tobacco at 4.5% (*n* = 35) (Figure 1). When taken together, the sweet flavours (fruit and sweet) accounted for 83.8% of all the most popular flavours.

### 3.6 Analysis of the Total Sample of Compliant Products

Similarly, analysis of the full sample of compliant flavours (non‐compliant flavours removed) showed the popularity of sweet flavours (9521 descriptors were identified for the 5145 compliant products). With the majority, 68.9% fruit (*n* = 6559), followed by 10.0% sweet (*n* = 952), 8.6% mint or menthol (*n* = 819), 4.5% tobacco (*n* = 428) and 4.1% sour (*n* = 390). In combination, fruit and sweet flavours were the most commonly used descriptors, making up 78.9% of the sample, presented in flavours and colours that may appeal to young people. Other categories made up less than 4.0%.

## 4 Discussion

This study aimed to determine whether online e‐cigarette retailers in New Zealand complied with flavour name restrictions introduced into law in the 9 months prior to the analysis. The regulations reduced the number of flavour names to a list of 57 words, of which retailers can select up to two to describe each product (Table 1). These regulations were an attempt to limit flavour descriptions that appeal to youth and children. For example, candy and soda flavours were banned. We also wanted to understand what flavours were popular on the New Zealand e‐cigarette retail market.

Our study revealed widespread non‐compliance among retailers. Of the 22 retailers, only 4 were fully compliant with the regulatory standards for flavour descriptions. Given the vast number of product variants available, some degree of non‐compliance with the relabelling regulations was expected. However, a significant proportion (25.8%; *n =* 1794) of the 6939 flavoured e‐cigarette products were non‐compliant.

The most frequent breach (*n* = 1346, representing 75.0% of all violations) involved retailers displaying the previous, non‐compliant flavour name alongside the new, compliant one. The New Zealand regulations stipulate that ‘A variant name [and packaging] on a vaping product must describe the actual flavour of the vaping product using only 1 or 2 flavour names listed in Schedule 4A’ (Table 1). Despite these clear guidelines, a significant proportion of e‐cigarette products on the New Zealand online market were advertised in this manner. A primary reason for the regulations was to prevent the use of words with obvious youth appeal, such as ‘candy’ and ‘soda’. The continued use of these flavours suggests that retailers value flavours with youth appeal (e.g., 17.6% of the most popular flavours analysed were non‐compliant candy, soda and desserts). This finding also suggests a lack of regulatory enforcement.

Similarly, the continued use of the word ‘ice’ to describe the menthol cooling sensation indicates the importance of this additive. The New Zealand Ministry of Health states that cooling agents may be used in products but cannot appear in the flavour name. It states that ‘ice’, along with other cooling descriptors, ‘are not recognised as flavours and cannot be used in product names’. Menthol has been used widely in traditional cigarettes to make tobacco more palatable to consumers; the strategy has been particularly effective in attracting young people [24]. Further, menthol has been used as a tool to target marginalised communities, and may therefore increase health inequities [25]. It appears that New Zealand retailers understand that continuing to market so‐called ‘ice’ products is an important way to attract consumers and maintain dependence [26].

Our analysis shows that the online e‐cigarette market in New Zealand is dominated by sweet and fruity flavours, which are particularly popular among youth [3]. Previous research shows that the most common flavours on the New Zealand online market prior to any regulations were fruity [16]. The extensive approved list of fruit flavours enables the industry to capitalise on sweet flavours that may appeal to youth. A total of 26 permitted fruit flavours and 7 additional sweet descriptors (caramel, chocolate cream, custard, honey, sweet, and vanilla) enable the industry to market 528 unique sweet combinations. Combinations such as Strawberry Cream and Sweet Raspberry, which are permitted under the regulations, may continue to have strong youth appeal, in flavours similar to those of many youth‐oriented products, such as candy and soda. Further, product packaging may also contribute to product appeal through a range of bright, attractive colours and styles.

Our study shows that despite a large number of permitted flavour combinations, retailers regularly flout the restrictions. This research also highlights that policies must be enforced; otherwise, they are, in effect, little more than a voluntary code of practice. However, flavour restrictions have been shown to be effective in other jurisdictions. An evaluation of the Californian law, which prohibits flavoured e‐cigarettes, found a reduction in the sale of e‐cigarette products compared to states without a flavour ban [27]. In another example, authorities in the Netherlands restricted e‐cigarette flavours only to tobacco and prescribed specific ingredients that can be used in the liquids [11]. This approach prevents the industry from using ‘compliant’ labels to disguise ingredients added to appeal to youth, as seen in other jurisdictions [28].

The findings of this study highlight the dominance of sweet and fruity flavours on the market. Sweet flavours play a key role in experimentation and initiation of vaping among young people [2]. Evidence suggests that sweet flavours are preferred by young people and reduce perceptions of harm [3, 29]. Reducing or removing sweet and fruity flavours from the market alongside strong enforcement may be an effective way to reduce e‐cigarette appeal to youth. Prior research has identified various strategies used by the e‐cigarette industry to exploit regulatory loopholes to protect the appeal of products in New Zealand [30, 31], Australia [32] and globally [33]. E‐cigarette policies need to be robust, comprehensive and pre‐empt industry workarounds.

A limitation of this study is that we examined only online retailers; physical retailers may stock different products. To determine the most popular flavours, we sorted websites by ‘best‐selling’ or ‘popular’; however, these metrics are unverified and can be manipulated by retailers to push certain products. However, the wider product sample shows proportions similar to those of the most popular flavours promoted by retailers, indicating broad consistency in the prominence and popularity of sweet flavours. The e‐cigarette market is dynamic, with rapidly evolving products to meet consumer demand. The data was collected in October—December 2024, and therefore, the market may have changed, although it is likely that fruit and sweet flavours remain popular [16]. Although we aimed to include the most popular retailers in our study, there are further online retailers operating in the New Zealand market that were not captured with our search strategy. Finally, as this study was conducted in New Zealand, it may not reflect the e‐cigarette markets in other countries.

## 5 Conclusion

Our analysis revealed widespread non‐compliance, with a significant proportion of products (25.8%) breaching the regulations. Retailers actively sought to bypass the regulations by displaying the previous, now‐prohibited flavour names alongside compliant ones. The persistent use of descriptors with youth appeal, such as ‘candy’, ‘cola’ and ‘ice’, demonstrates the importance of these flavours in product appeal.

This research also highlights the weakness of the regulations. By permitting a broad list of sweet and fruit flavour names, the policy effectively allows the industry to continue capitalising on combinations appealing to youth. The finding that sweet and fruity flavours account for 78.9% of all products indicates that a policy that targets only flavour names, rather than flavours themselves, is insufficient to address the core problem of e‐cigarettes' appeal to youth. For public health policy to succeed, it must be strong enough to pre‐empt industry workarounds and be accompanied by rigorous enforcement.

## Funding

This research was supported by the University of Auckland Faculty of Medical & Health Sciences.

## Ethics Statement

The authors have nothing to report.

## Conflicts of Interest

The authors declare no conflicts of interest.

## Supporting information


**Table S1:** Full list of retailers included in the sample.

## Data Availability

The data that support the findings of this study are available from the corresponding author upon reasonable request.
