# Supplementary material for: Restricting E‐Cigarette Flavour Names to Reduce Youth Appeal: An Analysis of New Zealand's 2024 Regulations
Source: Health Promot J Austr. 2026 Mar 16;37(2):e70171. doi: 10.1002/hpja.70171 (PMC12991852; doi:10.1002/hpja.70171)
Supplement: Supplementary file 1 — Table S1: Full list of retailers included in the sample. [file HPJA-37-0-s001.docx]

**Supplementary Table 1**

| Retailer Name | Retailer Website |
| --- | --- |
| VAPO | www.vapo.co.nz |
| Vuse | www.vuse.co.nz |
| Shosha | www.shosha.co.nz |
| Podlyfe | www.podlyfe.co.nz |
| Vape Merchant | www.vapemerchant.co.nz |
| NZVAPOR | www.nzvapor.com |
| Vapourium | www.vapourium.nz |
| Cosmic NZ | www.cosmicnz.co.nz |
| getalt | www.getalt.co.nz |
| Big Vape | www.bigvape.co.nz |
| Mixology Vape | [www.mixologyvape.co.nz](http://www.mixologyvape.co.nz/) |
| Puffing Kiwi | [www.puffingkiwi.co.nz](http://www.puffingkiwi.co.nz/) |
| The Vape Shop | [www.thevapeshop.co.nz](http://www.thevapeshop.co.nz/) |
| IGET | [www.nzvapez.co.nz](http://www.nzvapez.co.nz/) |
| Vapeys NZ | [www.vapeys.co.nz](http://www.vapeys.co.nz/) |
| RELX | [www.relxnow.co.nz](http://www.relxnow.co.nz/) |
| Kiwicig | www.kiwicig.co.nz |
| The Vaping Kiwi | [www.thevapingkiwi.co.nz](http://www.thevapingkiwi.co.nz/) |
| Vape2Go | [www.vape2go.co.nz](http://www.vape2go.co.nz/) |
| Vape Traders NZ | [www.vapetraders.co.nz](http://www.vapetraders.co.nz/) |
| Vapoureyes | www.vapoureyes.co.nz |
| IQOS | [www.nziqos.com](http://www.nziqos.com/) |
